# Athletic Burnout and Its Association with Diet in Children and Adolescents

**DOI:** 10.3390/life13061381

**Published:** 2023-06-13

**Authors:** María Morales-Suárez-Varela, Isabel Peraita-Costa, Agustín Llopis-Morales, Agustín Llopis-González

**Affiliations:** 1 Research Group in Social and Nutritional Epidemiology, Pharmacoepidemiology and Public Health, Department of Preventive Medicine and Public Health, Food Sciences, Toxicology and Forensic Medicine, Faculty of Pharmacy, Universitat de València, Av. Vicent Andrés Estelles s/n, 46100 Burjassot, València, Spain; isabel.peraita@uv.es (I.P.-C.); allomo@alumni.uv.es (A.L.-M.); agustin.llopis@uv.es (A.L.-G.); 2 CIBER of Epidemiology and Public Health, Carlos III Health Institute, Av. Monforte de Lemos 3-5 Pabellón 11 Planta 0, 28029 Madrid, Madrid, Spain

**Keywords:** burnout, Mediterranean diet, nutrition, kidmed, child, adolescent, athlete, sport, performance, activity

## Abstract

Children today are constantly exposed to several risk factors and high levels of stress that can impact their mental, emotional, and physical health, which can trigger burnout. The objective of this study was to determine the prevalence and frequency of burnout in young amateur athletes and to study the role of the Mediterranean diet on burnout risk. An observational, cross-sectional, and descriptive study of 183 basketball players between 8 and 15 years old was carried out. Adherence to the Mediterranean diet was assessed using the KIDMED questionnaire and the risk of burnout was assessed with the Athlete Burnout Questionnaire. Medians, minimums and maximum values for quantitative variables and absolute frequencies and percentages for qualitative variables were obtained. The results show a higher percentage of burnout among girls. The children who meet the established threshold for burnout spend more time watching television. Participants with better adherence to the Mediterranean diet have lower burnout values in both genders and those with a higher risk of burnout have a worse adherence to the Mediterranean diet. Therefore, it is important to implement a balanced diet appropriate to the individual needs of the athlete.

## 1. Introduction

In Spain, between 58% and 71% of children between 6 and 15 years old participate in some extracurricular sport or training at least once a week [1], which can affect their emotional and psychological state. It is necessary to motivate both young people and their families to understand the benefits that sport brings to their health as this is necessary to prevent future psychological problems, such as burnout syndrome (BS) [2,3] which appears in the 10th and 11th International Classification of Diseases (ICD-10 and ICD-11) with the codes Z73.0 and QD85, respectively. 

In this context, there is a need to promote a general quality of life in the environment surrounding the child athlete, while taking into consideration that while extracurricular activities can be beneficial, the well-being of these children can be negatively affected by overscheduling their lives. 

The American and Canadian Association of Dietetics and the American College of Sports Medicine suggested in their position paper of 2016 that physical activity, sports performance, and recovery of the child and/or adolescent athlete can improve with proper nutrition [4]. This is also reflected in the Spanish Pediatric Association’s recommendations for the athletic child [5]. 

Several studies have highlighted the potential health benefits offered by adherence to a balanced and varied diet that fully meets the pattern of the Mediterranean Diet (MD) [6,7,8,9,10]. The MD is characterized by using olive oil as the main additional fat, the consumption of fruits and vegetables, nuts, cereals, and legumes (as the main source of dietary fiber), fish, and the occasional consumption of red meat. 

While traditionally associated with adults who are exhausted and disillusioned with their jobs, BS has now spread from the workplace to youth sports. The most widely accepted definition of BS in the context of sports practice is that formulated by Raedeke, which was adapted from the classical conceptualization of Maslach and Jackson (1981; 1984) [11,12,13,14]. Burnout in the context of sports practice can be defined as a syndrome of physical/emotional exhaustion, sport devaluation, and reduced athletic accomplishment [15]. Emotional and physical exhaustion is associated with the feeling of fatigue from intense training and competition [15]. The feeling of inefficacy that may develop when athletes cannot achieve personal goals or perform below expectations is associated with a reduced sense of accomplishment [15]. Finally, sport devaluation refers to losing interest in the sport or the feeling of resentment towards the sport [15].

The appearance of BS in the context of youth sports may be in part due to the increasingly competitive nature of these. It is considered a reaction to the chronic stress suffered by young athletes when they are unable to cope with the demands associated with the sport and any other demands such as those related to school or family obligations. The pressure that leads to the appearance of BS can stem from external or internal sources. Parents who pressure their child, family life that revolves around sport and negative coaching behaviors are some of the possible external factors. Meanwhile, in some athletes, personality characteristics, such as perfectionism, act as internal factors that make them vulnerable to BS.

Athlete BS can lead to a variety of adverse outcomes such as affective problems (low mood and hostility towards sport-related activities); cognitive issues (loss of focus, memory, and helplessness); physical aspects (fatigue, increased probability of injury); and behavioral issues (absenteeism and poor sports performance) [15]. Young athlete BS can also negatively impact academic performance, affecting parent–child and peer relationships [16]. 

BS in sports has historically not aroused the same interest for researchers. Nowadays, however, BS is generating great concern in sports due to its negative consequences for athletes [17,18,19]. Various investigations have studied these consequences at the cognitive, physiological, and behavioral levels. Burnout syndrome in an athlete can eliminate the reasons that led them to start and stay in the sport in the first place, leading to premature abandonment. Sports withdrawal and the consequent disruption of the one-dimensional identity established around the sport is one of the most worrisome consequences of BS [19,20,21]. The type of sports practice, stress, pressure exerted by the coach, teammates and family members, or excessive concern for body image can increase the risk of developing BS [21,22,23]. Given that some of the same risk factors described for a work/academic environment associated with BS exist even in amateur sports practice, it would be expected that BS is present in young amateur athletes.

Occupational BS and academic BS have been associated with negative dietary behaviors in several previous studies [24,25,26,27,28,29,30,31,32,33,34]. The existence of this association between BS and these behaviors suggests that the possible presence of BS could be an important factor when explaining disordered eating patterns in individuals under other similar conditions of stress such as BS related to sports practice. Given the fact that similar conditions to those possibly related to the association between BS risk and diet may also be found in youth sports and that previous studies have associated BS with negative dietary behaviors, it would be reasonable to expect similar associations between BS and diet pattern to arise in the context of youth athletic practice.

All this leads us to think that there are sufficient arguments to ensure that the presence of BS in children and adolescents who practice some type of sports discipline is an objective fact, which may be associated with various external factors in their daily lives, such as diet.

The objective of the present study is to study the prevalence and determine the frequency of BS in athletic children, as well as to determine if there exists an association between BS with nutrition, through the adherence to MD, and lifestyle of the participants.

## 2. Materials and Methods

### 2.1. Study Design

This was an exploratory descriptive cross-sectional study conducted in Valencia (Spain) to determine the prevalence of BS in child and adolescent athletes and its association with MD adherence and sedentary lifestyle characteristics of the participants.

### 2.2. Participants and Recruitment

Currently there is no officially recognized and accepted definition for what characteristics define an athlete. However, several associations and authors have created their own definitions [35,36,37,38,39]. A previous publication [40] on the matter provided four criteria which need to be met simultaneously in order to fulfill the definition of athlete and that have been used in this study to identify “child athletes”:

- To be training in sports aiming to improve their performance or results;
- To be actively participating in sport competitions;
- To be formally registered in a local, regional or national sport federation as a competitor;
- To have sport training and competition as their major activity or focus of interest, almost always devoting several hours in all or most of the days to these sport activities, exceeding the time allocated to other professional or leisure activities.

The target population of this study was children and adolescents that met these definitions of athlete. This study consists of a convenience sample of healthy children and adolescents who were part of the Jovens L’Eliana Basketball Club. 

Recruitment began with the presentation to the sports club of a formal letter and the authorization of the project by the Department of Education, Research, Culture and Sports of the Generalitat Valenciana (2015/27896) and Ethics Committee of the Universitat de València. Subsequently, a meeting was arranged with the director and parents’ association to present the study. After receiving approval from both the sport club director and the parents’ association, the sports club was considered as a participating center. A letter with a brief description of the study was sent to all the parents or legal guardians of the children and adolescents of the club (*n* = 218) with informed consent attached. Only after the signed informed consent was returned was the child or teen included in the study. 

**Figure 1:**
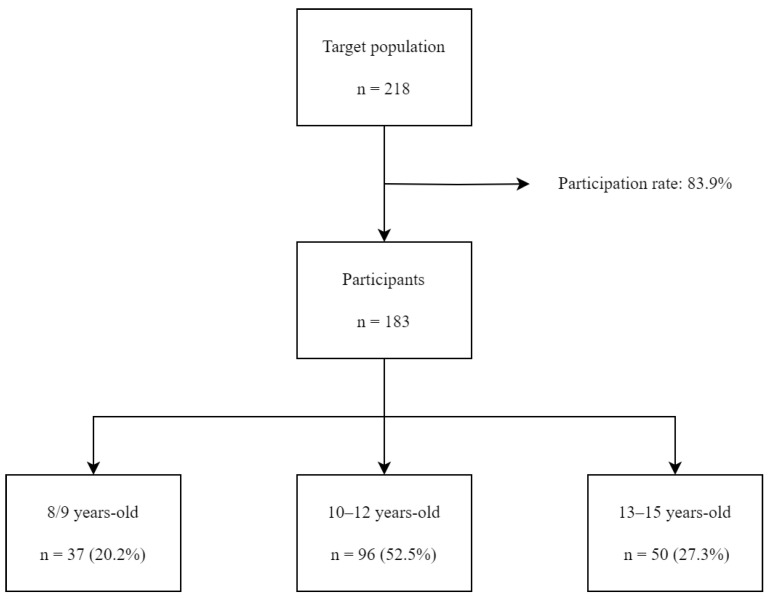
Flow diagram of the recruitment and enrollment of the studied sample.

A total of 183 healthy children and adolescents were finally included. The sample consisted of 107 (59%) boys and 76 (42%) girls. The age of the participants ranged from 8 years old to 15 years old (Figure 1). 

### 2.3. Instruments, Measures and Procedures

Participants and/or parents or legal guardians were interviewed using a questionnaire to obtain information about the participant’s age, gender, medical history, medication, and special dietary restrictions. Parents or legal guardians were also asked to report their child’s weekly frequency of technology use, such as the hours spent watching TV, hours spent playing video games and if the child had their own mobile phone. 

#### 2.3.1. Nutritional Assessment

The dietary intake was evaluated using the KIDMED questionnaire (Quality Index of the Mediterranean Diet for children, adolescents and young people) [41]. The KIDMED questionnaire is one of the most used scoring systems to assess adherence to MD in minors [41,42,43]. The questionnaire consists of 16 questions that must be answered in an affirmative/negative manner (yes/no). There are 12 questions that present a positive connotation (consumption of olive oil, fruits, vegetables, legumes, cereals, pasta or rice, fish and dairy products) and 4 questions that present a negative connotation (consumption of fast food, pastries, sweets and lack of breakfast). Questions with a positive connotation score +1, while questions with a negative connotation score −1. According to the KIDMED, the index can vary between 0 (minimum adherence) and 12 (maximum adherence).

#### 2.3.2. Assessment of Burnout Syndrome

The risk of BS was evaluated using the ABQ (Athlete Burnout Questionnaire) developed by Raedeke and Smith [44,45], a sport-specific adaptation of the Maslach Burnout Inventory [46] including several items from the Eades Athlete Burnout Inventory [47]. The ABQ is by far the most widely used questionnaire for assessing athlete burnout symptoms [48] and has been translated into several languages, including Spanish [49]. The ABQ has shown adequate psychometric properties with different athlete populations [50,51].

The ABQ survey was completed by young people and adolescents, under the supervision of the face-to-face pollster. For this study we used the reduced version of the Spanish version prepared by Arce et al. [14] constituted of 15 items, 5 for each of the three dimensions studied: physical/emotional exhaustion (PEE), reduced sensation of achievement (RSA) and devaluation of sports practice (DSP). Each response had five possible options: “Almost never”, “Seldom”, “Sometimes”, “Often”, “Almost always”. All items are semantically anchored on a 5-point Likert-type scale ranging from 1 (Almost never) to 5 (Almost always), and means are calculated to obtain subscale scores. 

The items are enunciated in such a way that the greater the numerical response of the athlete, the greater the experience of BS experienced, except for items 1 and 14, which are formulated in the opposite direction, i.e., the lower the numerical response, the greater the degree of BS. These items were inverted before calculating the total and subscale mean scores; therefore, a higher score means a higher risk of BS.

#### 2.3.3. Data Collection

The data collection took place between the months of April to June 2017. The data were collected in a group before a training session. Since the study participants were minors, the parents or legal guardians were duly informed about the purpose of the study and the protocol for collecting information. The questionnaires of extra-sports habits and KIDMED in the case of children aged 8 to 12 years were filled in by the parents so as not to generate biases in the answers. The ABQ was completed exclusively by the athlete in all age groups. The questionnaire was completed with the trained interviewer present at the time the children were completing it. The interviewer led the children through the questionnaire explaining each item and answering any questions or doubts the children had.

### 2.4. Data Analyses

The results are expressed in frequency and percentages for categorical data. To verify the association between the occurrence of BS and categorical variables, we applied Student’s t-test or Pearson’s chi-squared test. To describe the quantitative characteristics of the sample, the normal distribution of data was tested using the Shapiro–Wilks test. The comparison between the groups, the BS group and the non-BS group, was tested using the Mann –Whitney U test or the Kruskal–Wallis H test for non-parametric data. The strength of the association between the variables was evaluated by means of the odds ratio (OR) with the corresponding confidence interval (CI). The margin of error used in the statistical test decisions was 5% (*p* < 0.05) and the confidence level was 95%. Statistical analysis was performed using the IBM SPSS software version 26 (SPSS Inc., Chicago, IL, USA).

## 3. Results

All participating children and adolescents were formally registered in the local basketball federation as competitors representing the Jovens L’Eliana Basketball Club. They spent 2 h twice a week (4 h/week) training in order to improve their performance. They participated in formal competitions at least once a week. For the participants, basketball was their main extracurricular activity and the one they devoted the most time to but not necessarily the only one they participated in. 

All participating children and adolescents resided within the same metropolitan area (Valencia), no significant differences were observed in family income level (middle class), and all participants spent almost the same amount of time daily at school (between 5 and 6 h). 

In the sample of 183 participating children and adolescents (107 (59%) boys and 76 (42%) girls) 8 years old to 15 years old, the prevalence of BS was 69%. The percentage of girls with BS was higher (90%) compared to the percentage of boys (54%).

### 3.1. Characteristics and Lifestyle of the Participants

Table 1 reflects the personal characteristics and lifestyle of the participants in relation to the BS. The group that presents the highest frequency of BS among boys is the group of 8–9-year-olds (59%) while the 13–15-year-old group has the highest proportion of BS (67%). In girls, the 8–9-year-old group once again has the highest frequency (59%) but the 10–12-year-old group has the highest proportion (71%). The percentage of children with mobile phones is the same in those suffering from BS compared to those who do not (46%). It is observed that children who have BS devote more hours to television compared to those without burnout (54%, <1 h, 40%, 1 h, 6%, 2–3 h vs. 79%, <1 h, 18% 1 h, 0% 2–3 h), with significant differences (*p* < 0.001). 

### 3.2. KIDMED Questionnaire

Table 2 shows the results of each of the KIDMED questionnaire items based on the presence of BS. There is no statistical difference between the overall mean KIDMED scores within the genders or among them. Mean scores were between 8.71 ± 1.98 and 9.07 ± 1.48 and no child or teen scored low enough to be included in the poor adherence category. The mean scores are very close to the cutoff point (KIDMED score of 8) between medium MD adherence and optimal adherence. 

Significant differences for fruit consumption were found in girls, with those with BS consuming more fruit. However, regarding the daily intake of vegetables, consumption in both boys and girls with BS is lower (69% and 77% vs. 94% and 100%), with statistically significant differences. 

The vast majority of participants consume fish on a regular basis (>82% in all cases), and pulses more than once a week (>63%). In girls, there is a statistical difference in the consumption of fish among those with BS (82%) and those without BS (100%).

In general, children who do not suffer from BS comply with the regular intake of pasta or rice compared to those who do suffer BS (86% vs. 71%), with significant differences between girls with (59%) or without (75%) BS (*p* = 0.003) and between genders with BS (*p* < 0.001), there being fewer girls than boys (59% vs. 86%) who consume pasta or rice regularly. 

Most participants do not comply with the recommended weekly intake of nuts (the highest proportion being 43%) and the only significant difference appears once again between girls with (41%) or without (25%) BS. Olive oil is used for cooking in all participants’ homes.

The entire sample has breakfast daily. The majority of children consume cereal grains or by-products (>88%) and dairy products (>94%) in the first intake of the day, observing that in children with BS there is a greater proportion of industrial bakery consumption (45% and 29%, respectively). The only significant differences appear between girls with (88% cereal products and 94% dairy products) or without (75% cereal products and 100% dairy products) BS.

### 3.3. Dimensions of Burnout and Its Relation to Medium/Optimal MD Adherence

Table 3 shows that in boys there is an association between BS and the presence of physical/emotional exhaustion and a reduced sense of achievement (10 (6;14) and 8 (5;12) vs. 7 (5;8) and 5 (5;8) respectively, *p* ˂ 0.001). Regarding the girls, significant differences were also found in physical/emotional exhaustion and a reduced sense of achievement dimensions between both groups (12 (7;21) and 8.5 (1;14) vs. 7 (7;8) and 6 (5;6) respectively, *p* < 0.001). When comparing the sample as a whole, significant differences were found in all the BS dimensions between both the BS and non-BS groups. In the comparison between boys with BS and girls with BS, significant differences appear in the presence of physical/emotional exhaustion and a reduced sense of achievement dimensions, with girls having higher BS scores.

For each of the BS dimension sub scores, no consistent pattern with MD adherence is found. Statistically significant differences are found in the RSA (9 (5;11) vs. 7.5 (5;12), *p* = 0.05) dimension for boys with BS and in PEE (13 (9;21) vs. 12 (7;20), *p* = 0.03) for girls with BS. For the boys with BS, RSA sub scores were higher in those with medium MD adherence while girls with BS and medium MD adherence had higher PEE sub scores than those with optimal adherence. 

### 3.4. Odds Ratios of Burnout 

Table 4 presents the crude and adjusted odds ratios for BS and each of the three dimensions associated with MD adherence, sex, age, mobile phone, TV and videogames. In the crude model, a significant risk is associated with girls in total BS (ORc: 7.18; 95%CI: 3.14–16.39) and its three dimensions PEE (ORc: 7.89; 95%CI: 344–18.11), RSA (ORc: 3.38; 95%CI: 1.79–6.36) and DSP (ORc: 38.62; 95%CI: 8.81–169.20) which is maintained in the adjusted model (ORa: 8.78; 95%CI: 2.41–32.01) (ORa: 12.60; 95%CI: 3.13–50.70) (ORa: 3.44; 95%CI: 1.31–8.99) (ORa: 44.17; 95%CI: 4.81–405.72). The crude model also shows a risk associated with 10–12-year-olds for the DSP dimension (ORc: 7.90; 95%CI: 2.92–21.39) but it disappears after adjustment. TV use of more than one hour per day is shown to be a risk factor for total BS (ORc: 4.00; 95%CI: 1.86–8.63) and the PEE (ORc: 3.42; 95%CI: 1.64–7.11) and RSA (ORc: 2.49; 95%CI: 1.32–4.68) dimensions in the crude model and after adjustment the risk remains for total BS (ORa: 5.52; 95%CI: 1.61–18.85) and the PEE dimension (ORa: 5.99; 95%CI: 1.72–20.84).

## 4. Discussion

The stated objective is to study the prevalence of BS in athletic children and determine if there exists an association between adherence to MD and lifestyle. 

Approximately two-thirds (69%) of study participants were at risk of suffering from BS. The study of Vives and Garcés de los Fayos [52] on a Spanish population refers a similar prevalence of approximately 50% having suffered, at least once, one of the dimensions of BS. Likewise, Medina Mojena and García Ucha [53] studied a sample of Cuban athletes presenting risks of suffering BS between 50% and 70% in two of the dimensions. On the other hand, Pedrosa and García-Cueto [54] reported a prevalence of around 3% in a sample of Spanish participants, values similar to those presented by Sanchez-Alcaraz Martínez and Gómez-Mármol [55] in a sample composed of Spanish and Spain-based tennis players with a prevalence of about 5%. Isorna Folgar et al. [56] found no Spanish canoeists with BS but 10% of those studied showed a high risk of developing BS, while in Tutte and Reche [57] almost 5% of Spanish female hockey players presented BS and Olivares Tenza et al. [58] found a 3% prevalence of BS in a Spanish athlete population. These significantly large differences in the reported prevalence of BS could be due to sport-specific reasons. It would seem reasonable to expect a difference between individual and team sports depending on the level of competition. A higher frequency of burnout in individual sports might be expected as a result of higher demands on time and effort and given that social support and relationships in team sports may serve as a buffer [59,60,61]. However, no conclusions can be made without further studies focused on the sport-specific actors that may be responsible for the observed different prevalence of BS. 

Regarding the gender difference, it was observed that girls had a much higher level of BS compared to boys (90% vs. 54%). The odds ratio attributed to sex for BS or one of its dimensions also showed a much higher risk in girls. Sex is the most determining factor of those studied here. Several studies have shown that women have a greater tendency to suffer symptoms of BS and stress [62,63,64]. Jones and Cale [65] argue that this trend is because men have greater opportunities in the competitive universe and, therefore, lower level of stress. 

In the results of our study, there is no clear pattern associated with age and BS. Garcés de los Fayos et al. [66] observed that younger participants present lower scores of BS than older participants, with BS increasing with age and years of practice. However, other authors have found no statistically significant differences in the total scores or in any of the dimensions [67]. This could be due to a lack or loss of motivation, also as a consequence of negative emotional factors or high sports demands from very early ages.

Regarding the study of each dimension, we can observe that the physical/emotional exhaustion dimension presents a higher average and median score than the other two dimensions. This could occur because children may overvalue the physical effort they make and/or may be more affected by sleep-related disorders [68,69]. On the other hand, the devaluation of sports practice dimension has a low average and median score, probably because, as it is a study based on very young athletes, this dimension will not be as affected as the others may be.

Children with medium adherence to MD may have a higher risk of BS than children with an optimal adherence to MD; however, no consistent significant statistical differences were found in this study. This could be due in part to the fact that the diets of the participants were actually very similar; mean KIDMED scores and optimal adherence rates did not present any statistical difference among the studied groups, and therefore the differences derived from diet may be too small to show an effect in this case. The sample as a whole presented very similar diets and differences due to individual items were almost impossible to determine even if separated into optimal and medium adherence groups. It would be of interest to study an athlete group with deficient adherence to the MD to see if differences could be found between those with deficient and optimal diets and possibly establish if a deficit of certain micronutrients may be linked to the premature appearance of BS in children and adolescent athletes.

Fruits, along with vegetables, legumes, cereals, nuts and olive oil, are the basis of the pyramid of a balanced diet such as MD. A low consumption of any of these foods could cause the deficit of essential micronutrients and this could be related to an increased risk of developing burnout, affecting in the same way sports performance.

In this study, statistically significant differences for the sample overall were observed in adherence to MD and BS in relation to fruit, vegetable and pasta/rice consumption. Curiously, fruit intake was associated with suffering from BS while vegetable and pasta/rice intake was associated with not suffering from BS. If the responses to each item of the KIDMED questionnaire were analyzed separating the sample by sex, it can be seen that boys with and without BS had responses that were almost identical, while for girls the responses varied much more between those with and without BS. This shows that the dietary pattern for boys was the same regardless of if they suffer from burnout or not while in girls the groups with and without BS had marked different dietary intake patterns. Girls with BS consumed more fruit, grains at breakfast and nuts and less vegetables, fish, pasta/rice and dairy products at breakfast.

Due to the antioxidants, which are essential to neutralize free radicals, and the micronutrients which vegetables contain, it could be that a non-MD constitutes a greater risk of suffering BS. Some particular deficits of micronutrients are responsible for the increased risk of suffering psychological disorders [70,71,72]. In addition, it has been shown that the practice of sports increases free radicals, which tend to produce fatigue and injuries in muscles [73,74].

The main macronutrient of cereals/grains and derivatives (pasta, rice, bread, etc.) as well as legumes and potatoes are slowly absorbed carbohydrates, which allow adequate filling of glycogen deposits, which is very important before athletic competition. Due to the great importance of cereals in the child athlete both functionally and in regards to performance improvement, we would expect the results that were obtained. Consumption of these foods in the right proportions could prevent the appearance of BS.

A low consumption of nuts was observed overall in girls, a result consistent with a study carried out in young kayaking women [5]. Given the importance they have in the health of child and adolescent athletes, their low consumption could be related to a high risk of suffering BS.

The data collected in the study of Durá Travé, 2006 [75] on the nutritional analysis of breakfast and lunch in Spanish adolescents explains how there is a widespread trend in adolescents in our population to have breakfast of a glass of milk with cookies and/or pastries. This model moves away from what would be a healthy breakfast, with refined sugars and SFA predominating. The results of our study indicate that a higher consumption of these products could influence the risk of developing BS.

Considering the lifestyles of the participants, the majority of children with BS spend more hours on television and video games/computers compared to children without BS. However, it is also observed that there is no difference in mobile phone ownership between the groups. Burnout increases with time spent on screens, which can affect children specifically in the dimension of physical/emotional exhaustion. This fact could be influenced by the decrease in rest in children as they spend too many hours watching television, playing video games or surfing the internet.

## 5. Strengths and Limitations

The study had some limitations. The sample size that could be obtained for the study was small, which limited the power of the statistical analysis. The use of a convenience sample introduces a selection bias in the study and as it is a non-probabilistic technique which has associated weaknesses in regard to statistical rigor, the extrapolation of the results to the population is difficult. There may be relevant data fundamental to characterize a possible overtraining that would lead to BS missing from the study. The questionnaire on the food intake of the participants did not collect the exact amount of the food consumed daily. Burnout in sports is characterized by the abandonment of sports practice, which makes it interesting in future studies to look for cases of abandonment of sports and if nutritional intake had an influence. There are no direct studies that relate diet with the appearance of BS in the pediatric population of athletes, and it may be interesting to continue with this research.

The strength of this study lies in its validity and reliability given the low dropout rate of the participants. The relatives and coaches of the participants showed interest in the study from the beginning and were helped at all times to complete the records. In addition, all study participants were followed during the same period of time. The questionnaires used (ABQ, KIDMED) are the instruments most used both to measure BS in athletes and to measure adherence to MD in populations of this age. This generates confidence that these questionnaires accurately and reliably evaluate adherence to MD and BS. However, it must be noted that the ABQ is designed for use in those 12 years of age or older which may introduce a selection bias in this study. Currently there is no tool validated for use in children that allows the identification of BS; therefore, the ABQ, which is validated in Spanish and in children from 12 years old and up, along with the lack of studies that identify it as not suitable for use in the 8–12-year-old age range, is the tool that best fits this study. This may be an important limitation; however, since there is no other more appropriate tool to identify this syndrome in this age range, it can be said that there is no evitable selection bias. 

## 6. Conclusions

In view of the results of this study, the prevalence of BS in children and adolescents can be estimated at around 69%, with girls presenting significantly higher rates than boys (90% vs. 54%). Even though, as a whole, there is no direct relationship between diet and the appearance of the BS, the deficit of certain micronutrients could be associated with the increased probability of having a premature onset of BS in children and adolescent athletes. No inference about the direction of the association can be made at this time. The inappropriate use of technology (mobile phones, TV, internet, etc.) could influence the exhaustion of the children/athletes. For all this, it would be advisable to perform a nutritional intervention from the first appearance of characteristic symptoms of BS, such as lack of motivation, low performance, or exhaustion of the athlete, to try to correct possible nutritional deficits that could occur or to check if this symptomatology is given by the lifestyle and/or demands associated with the sport, as well as planning an adequate nutritional program in a personalized way to cover the individual needs of the athlete according to the degree and intensity of the sport they perform, their gender, their body composition and their state of maturity.

## Figures and Tables

**Table 1:** Presence of burnout in relation to the sex and personal characteristics of the participants.

	Boys (*n* = 107)			Girls (*n* = 76)			Total (*n* = 183)			
	YES *n* = 58 (54%)	NO *n* = 49 (46%)	*p*_1_	YES *n* = 68 (90%)	NO *n* = 8 (10%)	*p*_2_	YES *n* = 126 (69%)	NO *n* = 57 (31%)	*p*_3_	*p*_4_
**Age**			0.06			0.18			<0.001	<0.001
8–9 years old	34 (59%) (54%)	29 (59%) (46%)	1.0	12 (18%) (100%)	0 (0%) (0%)	-	46 (37%) (61%)	29 (51%) (39%)	0.076	<0.001
10–12 years old	4 (7%) (29%)	10 (20%) (71%)	0.047	40 (59%) (91%)	4 (50%) (9%)	0.63	44 (35%) (76%)	14 (25%) (24%)	0.180	<0.001
13–15 years old	20 (35%) (67%)	10 (20%) (33%)	0.09	16 (24%) (80%)	4 (50%) (20%)	0.12	36 (29%) (72%)	14 (25%) (28%)	0.577	0.18
**Mobile phone**	24 (41%) (57%)	18 (37%) (43%)	0.62	34 (50%) (81%)	8 (100%) (19%)	0.02	58 (46%) (69%)	26 (46%) (31%)	0.96	0.31
**TV**										
<1 h/day	30 (52%) (43%)	39 (80%) (57%)	0.003	38 (56%) (86%)	6 (75%) (14%)	0.51	68 (54%) (60%)	45 (79%) (40%)	<0.001	0.64
1 h/day	26 (45%) (71%)	10 (20%) (29%)	0.008	24 (36%) (100%)	0 (0%) (0%)	0.10	50 (40%) (83%)	10 (18%) (17%)	0.003	0.28
2–3 h/day	2 (3%) (100%)	0 (0%) (0%)	<0.001	6 (9%) (100%)	0 (0%) (0%)	0.006	8 (6%) (100%)	0 (0%) (0%)	0.12	0.39
**Videogame**										
˂1 h/day	27 (47%) (59%)	19 (39%) (41%)	0.42	20 (29%) (100%)	0 (0%) (0%)	0.17	47 (37%) (71%)	19 (33%) (29%)	0.61	0.05
1 h/day	26 (45%) (52%)	24 (49%) (48%)	0.67	10 (15%) (83%)	2 (25%) (17%)	0.81	26 (29%) (62%)	16 (46%) (38%)	0.24	<0.001
2–3 h/day	0 (0%) (0%)	2 (4%) (100%)	<0.001	2 (3%) (100%)	0 (0%) (0%)	0.24	2 (2%) (50%)	2 (4%) (50%)	0.78	0.73
>3 h/day	0 (0%) (-)	0 (0%) (-)	-	2 (3%) (100%)	0 (0%) (0%)	0.24	2 (2%) (100%)	0 (0%) (0%)	0.002	-
**Medication**	0 (0%) (-)	0 (0%) (-)	-	2 (3%) (100%)	0 (0%) (0%)	0.24	2 (2%) (100%)	0 (0%) (0%)	0.002	-
**MD (Optimal)**	44 (76%) (54%)	38 (78%) (46%)	0.81	56 (82%) (90%)	6 (75%) (10%)	0.63	100 (79%) (69%)	44 (77%) (31%)	0.76	0.37

Values are presented as *n* (%). Percentages were calculated by both columns and rows. *p* was calculated with Student’s *t*-test or Pearson’s chi-square test (significance was *p* < 0.05). *p*_1_: boys with BS vs. without BS. *p*_2_: girls with BS vs. without BS. *p*_3_: children with BS vs. without BS. *p*_4_: boys with BS vs. girls with BS.

**Table 2:** Mediterranean diet adherence according to sex and burnout.

	Boys (*n* = 107)			Girls (*n* = 76)			Total (*n* = 183)			
KIDMED Item	YES *n* = 58 (54%)	NO *n* = 49 (46%)	*p*_1_	YES *n* = 68 (90%)	NO *n* = 8 (10%)	*p*_2_	YES *n* = 126 (69%)	NO *n* = 57 (31%)	*p*_3_	*p*_4_
Daily fruit	54 (93%)	43 (88%)	0.54	58 (85%)	6 (75%)	0.02	112 (89%)	49 (86%)	0.57	0.16
Second daily fruit	24 (41%)	16 (33%)	0.35	34 (50%)	2 (25%)	0.03	58 (46%)	18 (32%)	<0.001	0.33
Daily vegetables	40 (69%)	46 (94%)	<0.001	52 (77%)	8 (100%)	<0.001	92 (73%)	54 (95%)	<0.001	0.34
Vegetables (>1/day)	26 (45%)	26 (53%)	0.40	44 (65%)	6 (75%)	0.07	70 (56%)	32 (56%)	0.94	0.03
Fish (≥2/week)	56 (97%)	41 (84%)	0.05	56 (82%)	8 (100%)	<0.001	112 (89%)	49 (86%)	0.57	0.01
Fast-food (>1/week)	0 (0%)	0 (0%)	-	0 (0%)	0 (0%)	-	0 (0%)	0 (0%)	-	-
Pulses (>1/week)	42 (72%)	31 (63%)	0.31	46 (68%)	6 (75%)	0.18	88 (70%)	37 (65%)	0.51	0.56
Pasta/rice (≥5/week)	50 (86%)	43 (88%)	0.81	40 (59%)	6 (75%)	0.003	90 (71%)	49 (86%)	0.03	<0.001
Cereal/grains breakfast	54 (93%)	47 (96%)	0.83	60 (88%)	6 (75%)	<0.001	114 (91%)	53 (93%)	0.78	0.35
Nuts (≥2/week)	21 (36%)	21 (43%)	0.43	28 (41%)	2 (25%)	0.005	49 (39%)	23 (40%)	0.85	0.57
Use of olive oil at home	58 (100%)	49 (100%)	-	68 (100%)	8 (100%)	-	126 (100%)	57 (100%)	-	-
Skips breakfast	0 (0%)	0 (0%)	-	0 (0%)	0 (0%)	-	0 (0%)	0 (0%)	-	-
Dairy breakfast	58 (100%)	47 (96%)	-	64 (94%)	8 (100%)	0.05	122 (97%)	55 (97%)	0.74	-
Processed food breakfast	23 (45%)	19 (39%)	0.53	20 (29%)	2 (25%)	0.41	46 (37%)	21 (37%)	0.97	0.07
Daily yogurt/dairy	17 (29%)	10 (20%)	0.29	24 (35%)	2 (25%)	0.07	41 (33%)	12 (21%)	0.11	0.47
Sweets (>1/day)	0 (0%)	0 (0%)	-	0 (0%)	0 (0%)	-	0 (0%)	0 (0%)	-	-
Total	9.07 ± 1.48	8.96 ± 1.54	0.71	8.71 ± 1.98	8.75 ± 2.05	0.96	8.87 ± 1.77	8.93 ± 1.60	0.83	0.26

Values are presented as *n* (%) or mean ± standard deviation. Percentages were calculated by columns. *p* was calculated with Student’s *t*-test or Pearson’s chi-square test (significance was *p* < 0.05). *p*_1_: boys with syndrome of BS vs. without BS. *p*_2_: girls with BS vs. without BS. *p*_3_: children with BS vs. without BS. *p*_4_: boys with BS vs. girls with BS.

**Table 3:** Dimensions of burnout according to MD adherence.

		Boys (*n* = 107)			Girls (*n* = 76)			Total (*n* = 183)			
		YES *n* = 58 (54%)	NO *n* = 49 (46%)	*p*_1_	YES *n* = 68 (90%)	NO *n* = 8 (10%)	*p*_2_	YES *n* = 126 (69%)	NO *n* = 57 (31%)	*p*_3_	*p*_4_
**Burnout**											
**PEE**		10 (6;14)	7 (5;8)	<0.001	12 (7;21)	7 (7;8)	<0.001	10 (6;21)	7 (5;8)	<0.001	0.03
**RSA**		8 (5;12)	5 (5;8)	<0.001	80.5 (1;14)	6 (5;6)	<0.001	8 (1;14)	5 (5;8)	<0.001	0.07
**DSP**		5 (5;7)	5 (5;5)	0.48	5 (3;9)	5 (5;5)	0.06	5 (3;9)	5 (5;5)	<0.001	<0.001
Total		22 (20;30)	17 (15;19)	<0.001	27 (21;39)	18 (17;19)	<0.001	25 (20;39)	17 (15;19)	<0.001	0.001
**Adherence to MD**											
**PEE**	Medium	9 (7;12)	7 (6;7)	<0.001	13 (9;21)	8 (8;8)	0.03	10.5 (7;21)	7 (6;8)	<0.001	0.04
	Optimal	10 (6;14)	7 (5;8)	<0.001	12 (7;20)	7 (7;7)	<0.001	10 (6;20)	7 (5;8)	<0.001	0.21
***p*_5_**		0.63	0.62		0.03	0.07		0.89	0.93		
**RSA**	Medium	9 (5;11)	5 (5;7)	<0.001	10 (7;12)	6 (6;6)	0.03	9.5 (5;12)	5 (5;7)	<0.001	0.68
	Optimal	7.5 (5;12)	5 (5;8)	<0.001	8 (1;14)	5.50 (5;6)	0.001	8 (1;14)	5 (5;8)	<0.001	0.03
***p*_5_**		0.05	0.88		0.73	-		0.11	0.79		
**DSP**	Medium	5 (5;5)	5 (5;5)	1.00	5 (5;8)	5 (5;5)	0.52	5 (5;8)	5 (5;5)	0.28	0.02
	Optimal	5 (5;7)	5 (5;5)	0.50	5 (3;9)	5 (5;5)	0.15	5 (3;9)	5 (5;5)	<0.001	<0.001
***p*_5_**		1.00	-		0.90	-		0.33	-		
Total	Medium	24 (20;25)	17 (16;19)	<0.001	28 (21;39)	19 (19;19)	0.02	25 (20;39)	17 (16;19)	<0.001	<0.001
	Optimal	21.5 (20;30)	17 (15;19)	<0.001	27 (21;38)	17.5 (17;18)	<0.001	25 (20;38)	17 (15;19)	<0.001	0.002
***p*_5_**		0.76	0.98		0.55	0.43		0.11	0.96		

PEE: Physical/emotional exhaustion; RSA: Reduced sense of achievement; DSP: Devaluation of sports practice. Values are presented as median (min; max). *p* were calculated with Mann–Whitney or Kruskal–Wallis test (significance was *p* < 0.05). *P*_1_: boys with BS vs. boys without BS. *p*_2_: girls with BS vs. girls without BS. *p*_3_: children with BS vs. children without BS. *p*_4_: boys with BS vs. girls with BS. *p*_5_: optimal MD adherence vs. medium MD adherence.

**Table 4:** Crude and adjusted odds ratios for burnout and dimensions according to MD adherence and personal characteristics of the participants.

		Burnout		PEE		RSA		DSP	
		ORc	95% CI	ORc	95% CI	ORc	95% CI	ORc	95% CI
**MD**	Optimal	Ref.	Ref.	Ref.	Ref.	Ref.	Ref.	Ref.	Ref.
	Medium	1.13	0.53–2.41	1.36	0.65–2.87	1.57	0.76–3.26	0.47	0.15–1.45
**Sex**	Male	Ref.	Ref.	Ref.	Ref.	Ref.	Ref.	Ref.	Ref.
	Female	7.18	3.14–16.39	7.89	3.44–18.11	3.38	1.79–6.36	38.62	8.81–169.20
**Age**	8/9 years old	Ref.	Ref.	Ref.	Ref.	Ref.	Ref.	Ref.	Ref.
	10–12 years old	1.98	0.92–4.29	1.26	0.60–2.60	1.27	0.62–2.58	7.90	2.92–21.39
	13–15 years old	1.62	0.75–3.50	1.44	0.66–3.14	2.06	0.98–4.34	1.09	0.29–4.10
**Mobile phone**	No	Ref.	Ref.	Ref.	Ref.	Ref.	Ref.	Ref.	Ref.
	Yes	1.02	0.54–1.90	1.13	0.60–2.11	1.56	0.86–2.85	0.96	0.44–2.08
**TV**	≤1 h	Ref.	Ref.	Ref.	Ref.	Ref.	Ref.	Ref.	Ref.
	>1 h	4.00	1.86–8.63	3.42	1.64–7.11	2.49	1.32–4.68	0.74	0.33–1.70
**Videogames**	≤1 h	Ref.	Ref.	Ref.	Ref.	Ref.	Ref.	Ref.	Ref.
	>1 h	0.90	0.16–5.07	0.25	0.04–1.43	2.50	0.44–14.05	2.31	0.40–13.23
		**Burnout**		**PEE**		**RSA**		**DSP**	
		**ORa**	**95% CI**	**ORa**	**95% CI**	**ORa**	**95% CI**	**ORa**	**95% CI**
**MD**	Optimal	Ref.	Ref.	Ref.	Ref.	Ref.	Ref.	Ref.	Ref.
	Medium	0.67	0.19–2.34	0.59	0.16–2.13	1.52	0.48–4.79	0.70	0.10–5.12
**Sex**	Male	Ref.	Ref.	Ref.	Ref.	Ref.	Ref.	Ref.	Ref.
	Female	8.78	2.41–32.01	12.60	3.13–50.70	3.44	1.31–8.99	44.17	4.81–405.72
**Age**	8/9 years old	Ref.	Ref.	Ref.	Ref.	Ref.	Ref.	Ref.	Ref.
	10–12 years old	3.01	0.24–38.53	0.13	0.01–2.39	0.52	0.09–3.12	2.57	0.29–22.41
	13–15 years old	7.91	0.44–142.82	1.51	0.09–24.60	1.97	0.24–16.22	0.85	0.05–14.37
**Mobile phone**	No	Ref.	Ref.	Ref.	Ref.	Ref.	Ref.	Ref.	Ref.
	Yes	0.57	0.08–4.19	0.57	0.07–5.07	0.79	0.15–4.14	0.62	0.08–4.80
**TV**	≤1 h	Ref.	Ref.	Ref.	Ref.	Ref.	Ref.	Ref.	Ref.
	>1 h	5.52	1.61–18.85	5.99	1.72–20.84	2.49	0.96–6.48	0.81	0.19–3.42
**Videogames**	≤1 h	Ref.	Ref.	Ref.	Ref.	Ref.	Ref.	Ref.	Ref.
	>1 h	0.23	0.02–3.26	0.05	0.00–0.75	1.42	0.10–19.94	1.46	0.07–30.52

PEE: Physical/emotional exhaustion; RSA: Reduced sense of achievement; DSP: Devaluation of sports practice. ORc: crude odds ratio; ORa: adjusted (MD, sex, age, mobile phone, TV, videogames) odds ratio.

## Data Availability

The data presented in this study are available upon reasonable request from the corresponding author. The data are not publicly available due to personal data protection.
